# Identifying Anti-Oxidant Biosynthesis Genes in Pearl Millet [*Pennisetum glaucum* (L.) R. Br.] Using Genome—Wide Association Analysis

**DOI:** 10.3389/fpls.2021.599649

**Published:** 2021-05-28

**Authors:** Chandra Bhan Yadav, Jayanti Tokas, Devvart Yadav, Ana Winters, Ram B. Singh, Rama Yadav, Prakash I. Gangashetty, Rakesh K. Srivastava, Rattan S. Yadav

**Affiliations:** ^1^Institute of Biological Environmental and Rural Sciences (IBERS), Aberystwyth University, Aberystwyth, United Kingdom; ^2^Chaudhary Charan Singh (CCS) Haryana Agricultural University, Hisar, India; ^3^International Crops Research Institute for the Semi-Arid Tropics, Patancheru, India; ^4^International Crops Research Institute for the Semi-Arid Tropics, Niamey, Niger

**Keywords:** pearl millet [*Pennisetum glaucum* (L.) R. Br.], antioxidant activity, phenolics, germplasm, genome-wide association study, marker-trait associations, candidate genes

## Abstract

Pearl millet [*Pennisetum glaucum* (L.) R Br.] is an important staple food crop in the semi-arid tropics of Asia and Africa. It is a cereal grain that has the prospect to be used as a substitute for wheat flour for celiac patients. It is an important antioxidant food resource present with a wide range of phenolic compounds that are good sources of natural antioxidants. The present study aimed to identify the total antioxidant content of pearl millet flour and apply it to evaluate the antioxidant activity of its 222 genotypes drawn randomly from the pearl millet inbred germplasm association panel (PMiGAP), a world diversity panel of this crop. The total phenolic content (TPC) significantly correlated with DPPH (1,1-diphenyl-2-picrylhydrazyl) radical scavenging activity (% inhibition), which ranged from 2.32 to 112.45% and ferric-reducing antioxidant power (FRAP) activity ranging from 21.68 to 179.66 (mg ascorbic acid eq./100 g). Genome-wide association studies (GWAS) were conducted using 222 diverse accessions and 67 K SNPs distributed across all the seven pearl millet chromosomes. Approximately, 218 SNPs were found to be strongly associated with DPPH and FRAP activity at high confidence [–log (*p*) > 3.0–7.4]. Furthermore, flanking regions of significantly associated SNPs were explored for candidate gene harvesting. This identified 18 candidate genes related to antioxidant pathway genes (flavanone 7-O-beta-glycosyltransferase, GDSL esterase/lipase, glutathione S-transferase) residing within or near the association signal that can be selected for further functional characterization. Patterns of genetic variability and the associated genes reported in this study are useful findings, which would need further validation before their utilization in molecular breeding for high antioxidant-containing pearl millet cultivars.

## Introduction

Pearl millet [*Pennisetum glaucum* (L.) R Br.] provides nutritious staple food grains worldwide especially to the poorest rural households in some of the hottest and driest rainfed farming regions of Africa and Asia. Global cereal production has been estimated to increase by ~1 billion tons over the last 50 years (FAOSTAT database, 2017). Pearl millet grown on 33 M ha worldwide is one of the four most important cereals grown in the tropical semi-arid regions of the world. It has excellent sustainability credentials, as the crop can easily survive on marginal lands, in harsh climatic conditions, and has a short growing season to complete its life cycle. It also is well-suited to multiple cropping systems under irrigated and dry land farming. It contains on average 92.5% dry matter, 2.1% ash, 2.8% crude fiber, 7.8% crude fat, 13.6% crude protein, and 63.2% starch, mostly of the resistant form, and high iron levels. It has a high energy content, lower readily available starch levels, higher fiber (1.2 g/100 g, most of which is insoluble), and 8–15 times greater α-amylase activity compared with wheat. Pearl millet also has a low glycemic index (50) and is gluten-free, thus, an ideal candidate grain for use in the functional-food market worldwide (Ali et al., 2003; Ragaee et al., 2006; Saleh et al., 2013). It also contains several phenolic compounds such as benzoic and cinnamic acid derivatives, anthocyanidins, flavonoids, lignans, and phytoestrogens, which play an important role in disease prevention (Muthamilarasan et al., 2016).

There are growing scientific evidences available, which confirm that diets, especially the plant food-based diets, rich in antioxidant compounds, offer a lower risk of developing cardiovascular diseases, certain kinds of cancers, and age-related degenerative processes (Pandey and Rizvi, 2009; Shahidi and Chandrasekara, 2013). Particular attention has been drawn to the role they play as “free radical scavengers,” which has provoked numerous studies into studying phenolic compounds in many plants, including cereals. The most studied sources of natural antioxidants are vegetables, fruits, and cereals (Khan et al., 2015). The presence of antioxidants in cereals is a consequence of the fact that all biological systems, including cereals, have a natural tendency to minimize the destructive potential of oxidation reactions, and consequently, they have developed their own multifunctional defense systems (Shahidi, 2000). Antioxidants found in cereals have the advantage of maintaining antioxidant capacity inside the human body following consumption (Serea and Barna, 2011). Cereals, especially millets, are the most commonly consumed foods in India and sub-Saharan Africa. They contain a wide range of phenolic compounds that are good sources of natural antioxidants (Ilesanmi and Akinloye, 2013). Antioxidant capacity of different cereal products, such as corn, wheat, rice, oats, and ready-to-eat breakfast cereals has been reported previously (Adom and Liu, 2002; Choi et al., 2007; Singh et al., 2012). In these cereals, antioxidant activity of different extracts correlated very well with their total plant phenolic content (Zieliński and Kozłowska, 2000). However, information on the extent of genetic variability and genes involved for antioxidant activities in pearl millet germplasm is scanty.

Rapid human population growth on a global scale is boosting the demand for a corresponding increase in crop grain yield, coupled with better nutritional credentials for a food secure future. Understanding the molecular and genetic control of useful traits, such as yield and nutritional quality, therefore, is becoming a major objective in the genetic study of staple cereal crops (Jin et al., 2010). Association mapping has been demonstrated as a powerful tool for dissecting genetic control of agronomic and other traits in plants (Yu and Buckler, 2006; Buckler and Gore, 2007; Zhu et al., 2008; Sehgal et al., 2015). Association panels today exist for a number of key crops (for example maize, wheat, sorghum, and pearl millet) that are routinely used in performing association analysis (Flint-Garcia et al., 2005; Maccaferri et al., 2006; Casa et al., 2008; Varshney et al., 2017). In pearl millet, an association panel known as pearl millet inbred germplasm association panel (PMiGAP) has been assembled from the world collection of pearl millet germplasm maintained at ICRISAT (Sehgal et al., 2015), which has recently been re-sequenced making available 28 million SNPs (Varshney et al., 2017) for use in genome-wide association studies. Availability of such resources is paving a powerful way for linkage disequilibrium (LD) mapping and association analysis of traits in pearl millet.

In the present study, we report on genetic variation for antioxidant-related traits in pearl millet, and the possible candidate genes associated with such traits, using genome-wide association studies.

## Materials and Methods

### Pearl Millet Germplasm Association Panel's Genotypes Used

A random set of 222 genotypes from within the pearl millet germplasm association panel (PMiGAP) was included in this study for antioxidant analysis (Supplementary Table 1). The PMiGAP is a collection of genotypes drawn from within the pearl millet core collection, landraces, cultivars, and breeding lines as explained in Sehgal et al. (2015) and represents geographical regions from 23 pearl millet growing countries across the world. The PMiGAP genotypes, thus, included in this study represent the entire global diversity of cultivated pearl millet. Further details of the origin of each of the PMiGAP genotypes used in the study are available in Sehgal et al. (2015) and Varshney et al. (2017). Seeds of each of the PMiGAP genotypes used for antioxidant determination were bulk multiplied by growing them in a uniform field condition following standard agronomic and seed multiplication procedure (Upadhyaya et al., 2008; Ramya et al., 2018). Each entry was planted in three rows by maintaining 15 cm between plants and 75 cm between rows. Field was applied with 100 kg/ha of DAP (di-ammonium phosphate) as basal dose; thinning was done to one plant per hill. Weeding was done two times during the seed multiplication plot. Each individual head was selfed before the emergence of panicle, and strict pollination was controlled to get the pure seeds of each line. Harvesting was done during physiological maturity. Threshing was done with Winterstieger single head thresher and cleaned to remove the remaining debris.

### Preparation of Extracts for Antioxidant Analysis From Pearl Millet Flour

Pearl millet flour was extracted for antioxidant analysis according to a modification of the method described in Akpanika et al. (2017). A quantity of washed grains (1 g) was milled in a customized robotic instrument (Labmann Automation, Middlesbrough, UK) to obtain a fine flour. Thirty milligrams of flour was extracted with 5 ml of 70% ethanol with intermittent stirring for 1 h. The extract was then centrifuged at 10,000 × *g* for 10 min at room temperature, and the supernatant was transferred to clean tubes. The pellets were then washed with 2.0 ml of 70% ethanol and centrifuged again at 10,000 × *g* for 10 min. The second supernatant fraction was added to the first to maximize recovery of target compounds. The ethanol was then removed using a heated centrifugal evaporator (Jouan RC10.22, Saint Herblain, France) set to 70°C. The dried extract was re-dissolved with 70% ethanol to an equivalent concentration of 15 mg/ml of the original sample and stored at 4°C until analysis. All biochemical analyses (see below) were carried out in triplicate, which allowed us to calculate the repeatability of these measurements.

### Determination of 1,1-Diphenyl-2-Picrylhydrazyl Radical Scavenging Activity

DPPH is a stable free radical for the determination of antioxidant or radical scavenging capabilities. The DPPH radical portion of the molecule is a centralized nitrogen atom that gives rise to a maximum absorbance at 515 nm in methanol in its oxidized form. When a solution of DPPH in its radical form is mixed with a proton-donating substance such as antioxidants, the radicals are scavenged, and DPPH=H is formed with a concomitant decrease in absorbance. The ability of the pearl millet ethanolic extracts to scavenge free radicals was determined against a very stable free radical DPPH (1,1-diphenyl-2-picrylhydrazyl). DPPH is violet in color, while the reduced product is colorless, and the loss of color was determined in a plate reader (Hauck, 2018). Aliquots of the sample extract (50 μl) at different concentrations were added to ethanolic solutions of 500 μl of DPPH (0.12 mg/ml). Each mixture was left for 20 min at room temperature in the dark. The absorbance was measured at 517 nm, and the activity was expressed as percentage of DPPH radical relative to control using the following equation: DPPH scavenging activity (%) = [(absorbance of control – absorbance of sample)/absorbance of control] × 100.

### Ferric-Reducing Antioxidant Power Assay

FRAP assay is a novel method for assessing antioxidant power where the ferric-reducing ability of a sample extract is tested. Ferric to ferrous ion reduction at low pH causes a colored ferrous–tripyridyltriazine complex to form. FRAP values are obtained by comparing the absorbance change at 593 nm in test reaction mixtures with those containing ferrous ions in known concentration (Benzie and Strain, 1999). The ferric-reducing antioxidant power (FRAP) assay measures the antioxidant potential of antioxidants to reduce the Fe^3+^/tripyridyl-s-triazine complex present in stoichiometric excess to the blue ferrous form (Hauck, 2018). FRAP reagent was freshly prepared by mixing together 10 mM 2,4,6-tripyridyl-s-triazine (TPTZ) and 20 mM ferric chloride in 0.25 M acetate buffer, pH 3.6 in proportions of 1:1:10 (v/v), respectively. Pearl millet extract (50 μl) was added to 1.5 ml of FRAP reagent. The absorbance was read at 593 nm after 4-min incubation at ambient temperature against distilled water as a blank in a plate reader. A calibration curve of ascorbic acid concentration (100–1,000 μmol/L) versus absorbance was used to calculate values, and results are expressed in mg/ml ascorbic equivalents/g dry weight for plant total extracts from three determinations.

### Sample Separation by High-Performance Liquid Chromatography and Statistical Analysis

Among the analyzed pearl millet samples, 40 samples were picked from the higher, lower, and mid-range antioxidant potential, and evaluated for total phenolic and flavonoid content by high-performance liquid chromatography with online photodiode array detection and electrospray ionization–ion trap tandem mass spectrometry (HPLC-PDA-ESI/MS^n^) analysis (Supplementary Table 2). Samples were prepared for analysis by solid phase extraction with Waters Sep-Pak C18 500 mg cartridges (WAT036945) prepared by passing through 4 ml of 100% methanol followed by 4 ml of purified water. The sample (500 μl) was then loaded onto the cartridge. The unbound aqueous fraction was eluted with 4 ml of purified water, and the bound fraction was then eluted with 4 ml methanol. Both fractions were collected in separate clean vials. The samples were then fully dried using a heated centrifugal evaporator at 13,000 rpm and 70°C, and reconstituted in 100 μl of 70% methanol. Secondary metabolites were analyzed by reverse-phase HPLC-PDA-ESI/MSn.

The analyses were carried out on a Thermo Finnigan LC-MS system (Thermo Electron Corp, Waltham, MA, USA) with a Finnigan PDA plus detector, a Finnigan LTQ linear ion trap with ESI source, and a Waters C18 Nova-Pak column (3.9 × 100 mm, particle size 4 μm). The column temperature was maintained at 30°C and equilibrated with 95% solvent A (water/formic acid 99.9:0.1 v/v) and 5% solvent B (methanol/formic acid 99.9:0.1 v/v) at a flow rate of 1 ml min^−1^, with 10% going to the mass spectrometer. Compounds were eluted by linear gradient to 65% solvent B over 60 min. Compounds were detected by PDA from 240 to 400 nm and MS in positive and negative mode from 150 to 1,500 m/z with the following MS parameters: sheath gas 30 and auxiliary gas 15 (both arbitrary units), spray voltage −4.0 kV in negative and 4.8 kV in positive ionization mode, capillary temperature 320°C, capillary voltage −1.0 and 45 V, respectively, and tube lens voltage −68 and 110 V, respectively.

Aglycones were identified by direct comparison with relevant flavonoid standards, and glycosylated flavonoid compounds were identified by comparison of UV spectra and MS2 fragmentation patterns with standard flavonoid compounds. Standards were purchased from Carbosynth Ltd. (Compton, Berkshire, UK).

### Statistical Analysis of Biochemical Traits Measured

The replicated mean data of 222 PMiGAP genotypes for the two traits *viz*., FRAP and DPPH expressed in mg/ml was subjected to one factor analysis of variance (ANOVA) using the statistical software OPSTAT to find out the significant differences among the PMiGAP lines for both FRAP and DPPH activity (Sheoran et al., 1998). The means along with critical difference and coefficient of variation values were calculated using the software OPSTAT (Sheoran et al., 1998). The correlations between phenolic compounds and antioxidant activities were statistically evaluated by two-tailed bivariate correlate analysis, and were indicated by Pearson's coefficient indices, and *p* < 0.05 was considered as statistically significant. Heritability in broad sense was calculated using OPSTAT software (Sheoran et al., 1998) as the ratio of genotypic variance (σ2g) to the phenotypic variance (σ2g) and was expressed in the form of percentage (Hanson et al., 1956). The multivariate principal component analysis was performed by the JMPv.8 software (SAS Institute, 2008) to compare the multivariate correlation for antioxidant activities and phenolic compounds.

### Genotyping and Filtering of Single-Nucleotide Polymorphisms

A set of SNPs resulting from 222 pearl millet accessions used in this study were obtained from ICRISAT (Varshney et al., 2017). The SNPs were filtered for site coverage (90%) and minimum minor allele frequency (MAF) of 0.01 with only bi-allelic markers using the Tassel ver. 5.2.64 software. Other SNP filtering criteria used in this study were no SSR motifs, no InDel marker, only bi-allelic SNPs, minor allele frequency (MAF) 0.01, and SNP quality score ≥30. Genetic variant annotation for SNPs and its effect on genes and protein were predicted using SnpEff (https://pcingola.github.io/SnpEff/). Low-quality SNPs and SNPs with missing data were removed using the protocols described in Iquira et al. (2015) and Kujur et al. (2015).

### Population Structure and Linkage Disequilibrium Mapping Analysis

The population structure analysis was performed using STRUCTURE software as described by Sehgal et al. (2015), which is based on Bayesian model-based cluster analysis (Pritchard et al., 2000). This was used for the assessment of patterns of genetic structure in 222 lines of the PMiGAP samples. This method used these 222 individuals to infer the fraction of an individual accession's genetic ancestry that belongs to a population, for a given number of populations (*K3–K10*). The “correct” K from the Ln probability of data [Ln P(D)], the delta-K values were estimated, as per the procedure suggested by Evanno et al. (2005). Maximum peak of ΔK was considered as the true cluster number.

The LD between pairs of polymorphic loci were analyzed using the software package TASSEL 5.2.64 (Bradbury et al., 2007; http://www.maizegenetics.net/) as per the instructions of the user manual. LD was estimated using the squared allele frequency correlations (*r*^2^), which is a measurement of the correlation between a pair of variables (Hill and Robertson, 1968). Decay of LD with genetic distance was estimated by nonlinear regression (SPSS Version 10.0) following the methods of Remington et al. (2001). The expected decay of LD was modeled as per Weir and Hill (1986).

### Genome-Wide Association Analysis

Marker-trait association (MTA) analysis was performed using TASSEL program, ver. 5.2.64 by two different models, generalized linear model (GLM) and mixed linear model (MLM) (Bradbury et al., 2007). The MLM model-based analysis essentially required the population structure (Q), kinship (K), and a total of 67 K high-quality SNPs from 222 pearl millet lines for generation of K-matrix (Yu et al., 2006). The K-matrix was generated using the default parameters by choosing “Centered_IBS” method to obtain a better estimate of additive genetic variance. The default settings of the program were used for filtering marker data for minimum genotype count and minor allele frequency. Furthermore, no compression option in combination with P3D′ for variance component estimation was adapted during MLM-based association analysis. The experiment-wise *p*-value provided a test of significance that corresponded to the experiment-wise error and was used to make decisions about the significantly associated markers. The markers were filtered at –log10 *p*-value ≥ 3.0 for considering as significant marker trait associations. The phenotypic variation (*R*^2^) explained in percentage was also recorded for each individual marker. MTA analyses were also performed with an additional statistical method using the principal component analysis (PCA) matrix using the GLM (Yu et al., 2006). A permutation test using 1,000 permutations was allowed to correct the *p-*value for multiple comparisons. Q–Q plots and Manhattan plots were generated using the R package qqman (https://cran.r-project.org/web/packages/qqman/index.html; Turner, 2014).

### Identification of Probable Candidate Genes

Significant SNPs from the GWAS were located by position and chromosome number on the *Pennisetum glaucum* reference genome (ftp://cegresources.icrisat.org/) using the CLC Genomics Workbench v.6.5 (CLC Bio, Aarhus, Denmark). Upstream and downstream regions surrounding each significantly associated SNP were searched in order to find and propose possible candidate gene. Furthermore, BLAST search of the NCBI database was conducted on each region of interest against the NCBI nr database.

## Results

### Antioxidant Capacity Analysis by 1,1-Diphenyl-2-Picrylhydrazyl Free Radical Scavenging Capacity

**Figure 1:**
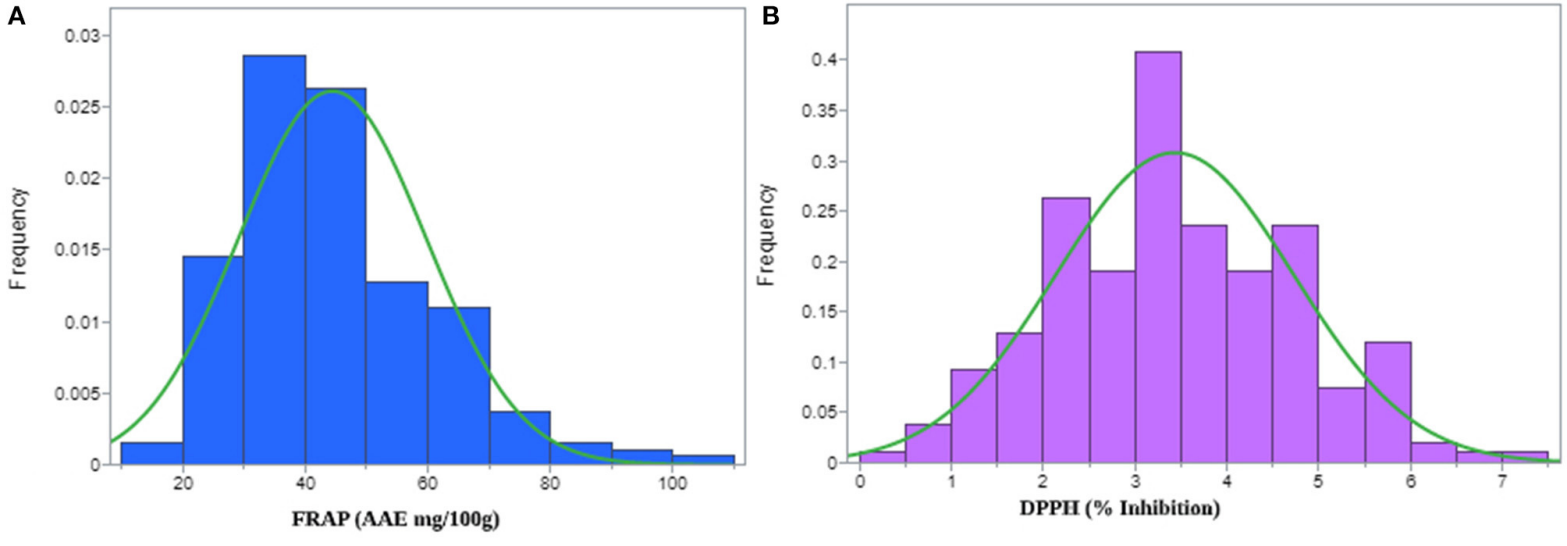
Histogram displaying normal distribution for **(A)** ferric-reducing antioxidant power (FRAP) and, **(B)** 1,1-diphenyl-2-picrylhydrazyl (DPPH) radical scavenging activities observed in the pearl millet inbred germplasm association panel (PMiGAP) of 222 genotypes.

DPPH free radical scavenging activities were assessed for the pearl millet flour extracts. Table 1 displays the significant differences in DPPH activity among the 222 genotypes of the PMiGAP. All the millet extracts scavenged DPPH radicals in a dose-dependent manner. DPPH scavenging activity (% inhibition) of the extracts ranged from 2.32% (in IP4965 genotype) to 112.45% (in IP10539 genotype) (Supplementary Table 3). Overall average DPPH scavenging activity of the extracts among pearl millet cultivars was found to be 53.75% (Figure 1A). These results indicated that extracts of pearl millet contain various phenolic antioxidants with the ability of donating hydrogen and scavenging free radicals. The heritability (measured as repeatability across three runs) was 99.92% for DPPH trait-regulatory factor and the phenotypic coefficient of variations for DPPH was 38.46.

**Table 1:** Analysis of variance (ANOVA) for phenotypic antioxidant traits [1,1-diphenyl-2-picrylhydrazyl (DPPH) and ferric reducing antioxidant power (FRAP)] measured in triplicate for 222 pearl millet genotypes of the pearl millet inbred germplasm association panel (PMiGAP) (one factor).

**Source of variation**	**Degree of freedom**	**Mean squares**		**F-calculated**	
		**FRAP (AAE^a^ mg/100 g)**	**DPPH (% inhibition)**	**FRAP (AAE mg/100 g)**	**DPPH (% inhibition)**
Genotypes	221	1,933.11	1,281.56	3,152.32^**^	3,758.92^**^
Error	442	0.61	0.34	-	-

** *p = 0.05*.

a *AAE, ascorbic acid equivalents*.

### Ferric-Reducing Antioxidant Power Assay

In this study, FRAP was calculated using the equation Y = 0.2453x, where x is the OD of the sample. The genotypic differences between the 222 genotypes were significant for FRAP activity (Table 1). Overall average FRAP activity of the extracts in pearl millet genotypes was 73.74 (AAE mg/100 g) (Figure 1B). The FRAP activity ranged from 21.68 mg/100 g (IP9446 genotype) to 179.66 mg/100 g (IP10579 genotype) of ascorbic acid (Supplementary Table 3). The highest ferric reducing ability of the extracts was found in IP7967 (150.57 mg/100 g) and IP9824 (149.27 mg/100 g). Higher antioxidant activity in these lines may be related to a higher content of phenolic compounds. The heritability of this extract (measured as repeatability across three runs) for FRAP was calculated, and it was found to be very high (99.91%). The phenotypic coefficient of variations for FRAP was recorded as 34.44.

### High-Performance Liquid Chromatography With Online Photodiode Array Detection and Electrospray Ionization–Ion Trap Tandem Mass Spectrometry Analysis

**Figure 2:**
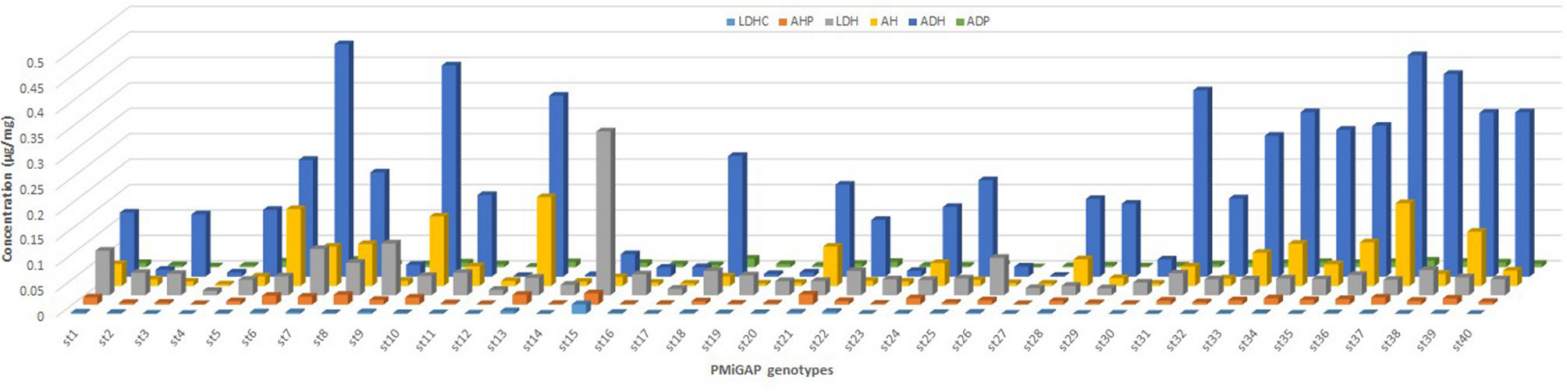
Flavonoid composition observed in selected 40 pearl millet germplasm samples. LDHC, luteolin-C-O-dihexosidecaffeate; AHP, apigenin-C-hexoside-C-pentoside; LDH, luteolin-C-O-dihexosidedihex; AH, apigenin-8-C-glucoside; ADH, apigenin-O-hexoside-C-hexoside C; ADP, apigenin-C-pentoside-C-pentoside.

**Figure 3:**
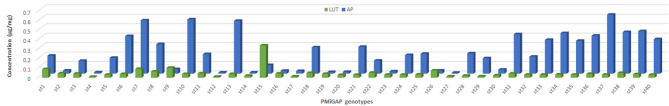
Distribution patterns for flavones fraction in 40 genotypes of the pearl millet germplasm. LUT, luteolin; AP, apigenin.

The total phenolics and flavonoid content that resulted from the HPLC-PDA-ESI/MS^n^ is presented in Figure 2, which shows the mass spectral data showing identified peaks in the representative chromatograms of the phenolic fractions of the selected millet grains. Phenolic compounds identified in millets were mainly of the flavonoids class, although spermidine hydroxycinnamte conjugates were also detected. Apigenin glycosides were the most prevalent flavones in the germplasm samples, and these included apigenin-8-*C*-glucoside (AH; Vitexin), apigenin-*C*-pentoside-*C*-pentoside (ADP), and apigenin-*O*-hexoside-C-hexoside (ADH). Luteolin-glycosides were also relatively abundant with luteolin-C-O-dihexosidedihex (LDH), and its caffeic acid conjugate was detected in extracts. The phenolamide, dicaffeoyl spermidine, was also observed in the sample extracts. An interesting pattern is seen in Figure 3, which demonstrates that apigenin is the predominant flavone in pearl millet samples with high levels of phenols, with the exception of Tift186, while for samples with lower total levels; apigenin is generally the major flavone core. The results also suggest that apigenin levels are far more variable than luteolin content. In millet phenolic extracts, several compounds from different flavonoid groups were detected that were either positively or tentatively identified as flavan-3-ol (monomers and dimers), flavonols, and their glycosides, and flavones, although in the current study, only flavone glycosides were found to be present in quantifiable levels.

### Correlation Studies Between Antioxidant Activities and Phenolic Compounds

**Figure 4:**
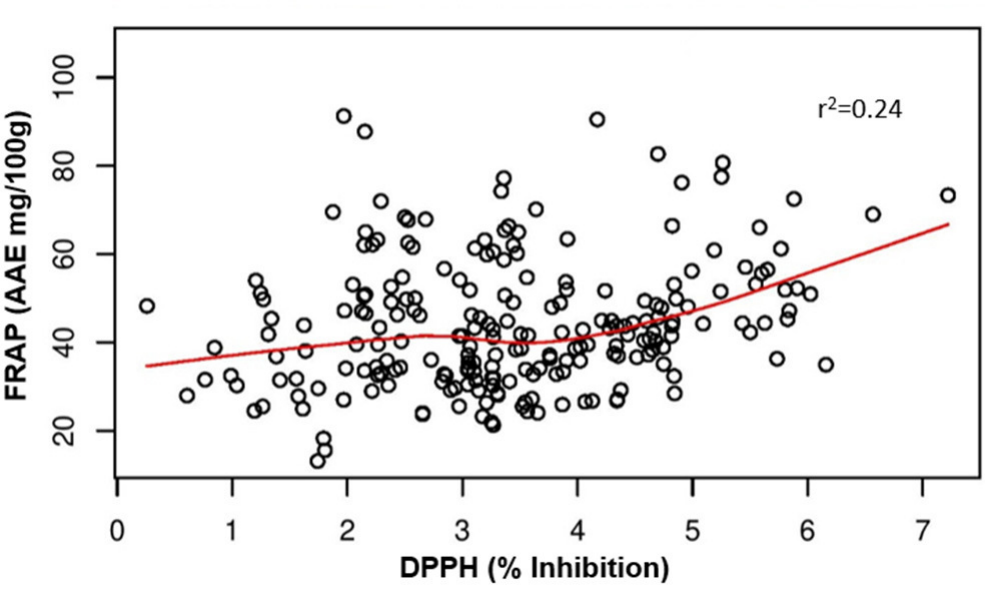
Correlation between FRAP and DPPH activities measured in the pearl millet inbred germplasm association panel (PMiGAP).

Polyphenols showed a positive and significant correlation with antioxidant activity (Table 2) as evident from the Pearson's coefficients between phenolics and different antioxidant activities. DPPH showed highly significant positive correlation with AHP, luteolin (LUT) (*r*^2^ = 0.88^*^), and FRAP (*r*^2^ = 0.64^**^) indicating a strong association between phenolic compounds and antioxidant activity. FRAP also showed significant and positive correlation with LHDC (*r*^2^ = 0.97^**^), and (LDH) (*r*^2^ = 0.96^**^). THL has also shown positive correlation with LDH (*r*^2^ = 0.92^**^) (Table 2). The results also clearly demonstrate a stronger correlation between total luteolin content and antioxidant activity (*r*^2^ = 0.88 and 0.79 with DPPH and FRAP assays, respectively) compared with total apigenin content (*r*^2^ = −0.39 and 0.49 with DPPH and FRAP assays, respectively) (Table 2). Multivariate principal component analysis (PCA), displayed the consistent dispersion pattern for each trait in relation to the first two principal coordinates. Based on measured eigenvalue, the first principal coordinate explained 39.2% of the total variation; the second coordinate could explain only 29.9%. The first five coordinates could explain a total of 96.01% variation. Thus, the first two components of the PCA explain a total of 69.07% of the variability among antioxidant activities and phenolic compounds (Supplementary Figure 1). The pairwise comparisons of DPPH and FRAP showed a weak correlation between the DPPH and FRAP with the *r*^2^ value as 0.24 (Figure 4). This indicates that the radical scavenging activity measured by DPPH does not correspond directly with the reducing antioxidant activity measured by FRAP.

**Table 2:** Correlation studies between phenolic compounds and antioxidant activities observed in selected 40 entries of the pearl millet germplasm.

	**DPPH**	**FRAP**	**LHDC**	**AHP**	**LDH**	**AH**	**ADH**	**ADP**	**AP**	**LUT**
DPPH	1	0.636^*^	0.572	0.880^**^	0.657	0.296	−0.491^**^	−0.420^**^	−0.392^*^	0.880^**^
FRAP		1	0.966^**^	0.792	0.965^**^	0.778	0.408	0.434	0.493	0.792
LHDC			1	0.546^**^	0.920^**^	0.074	−0.080	0.190	0.272	0.546^**^
AHP				1	0.501^**^	0.718^**^	0.540^**^	0.430^**^	0.762^**^	1.000^**^
LDH					1	−0.052	−0.052	0.212	0.286	0.501^**^
AH						1	0.724^**^	0.371^*^	0.788^**^	0.718^**^
ADH							1	0.653^**^	0.926^**^	0.540^**^
ADP								1	0.662^**^	0.430^**^
AP									1	0.762^**^
LUT										1

** *Correlation is significant at the 0.01 level (two tailed)*.

* *Correlation is significant at the 0.05 level (two tailed)*.

*LDHC, luteolin-C-O-dihexosidecaffeate; AHP, apigenin-C-hexoside-C-pentoside; LDH, luteolin-C-O-dihexosidedihex; AH, apigenin-8-C-glucoside; ADH, apigenin-O-hexoside-C-hexoside C; ADP, apigenin-C-pentoside-C-pentoside; AP, apigenin; LUT, luteolin*.

### Identification and Distribution of Single-Nucleotide Polymorphisms

A total of 67,979 high-quality SNPs distributed all over the seven chromosomes of pearl millet genome were identified (Supplementary Figure 2). In general, one SNP change was observed at every 23,015 bases in the genome. The average nucleotide change rate was 43.35 SNPs per 1,000 bases of the genome. The maximum SNP change rate was observed on chromosome 5, which was 46.74 SNPs per Mb region of the genome and minimum on chromosome 4 (38.04). Structural annotation of 67,979 SNPs revealed the presence of 4,027 (~1.3%) SNPs in exonic regions, followed by 57,251 (19.4%) in intergenic regions. A total of 2,177 SNPs (0.73%) showed non-synonymous types of modification, whereas 1,831 (0.62%) were synonymous SNPs. A total of 9,605 (3.25%) SNPs were underlying from intergenic region, and 294 (0.003%) SNPs were present in 5′ UTR regions (Supplementary Figure 3).

### Population Structure and Linkage Disequilibrium Analysis

The clustering program has estimated the membership probability (Q-matrix) of each PMiGAP accession to combine into a number of hypothetical subpopulations (K3–K10), and ΔK value was generated for subsequent runs. The optimum K at run 6 showed maximum peak during cluster analysis. In this way, Bayesian model-based cluster analysis revealed that the 222 individual genotypes were clustered into six groups (*K* = 6) (Supplementary Figure 4). These individuals were further classified into the ones with “pure” ancestry (where >50% of their inferred ancestry was derived from only one of the clusters) and “mixed ancestry” or “admixtures” (where <50% of inferred ancestry was derived from more than one cluster). The majority of the accessions belonged to the “mixed” ancestry, and the remaining 80 individuals from all six clusters were of “pure” ancestry.

The extent of LD was assessed among 67,979 pairs of loci (Supplementary Figure 5). Across all accessions, 2.08% of the total marker pairs showed a significant level of LD (*P* < 0.01). The average of r^2^ for all pairs was 0.108, which may be attributable to low levels of markers. Counts for individual *r*^2^ values were also analyzed by Varshney et al. (2017), where the *r*^2^ threshold was set as 0.2, and rapid LD decay of <0.5 kb in PMiGAP lines (84–444 bp) was observed.

### Markers Associated With 1,1-Diphenyl-2-Picrylhydrazyl and Ferric-Reducing Antioxidant Power

**Figure 5:**
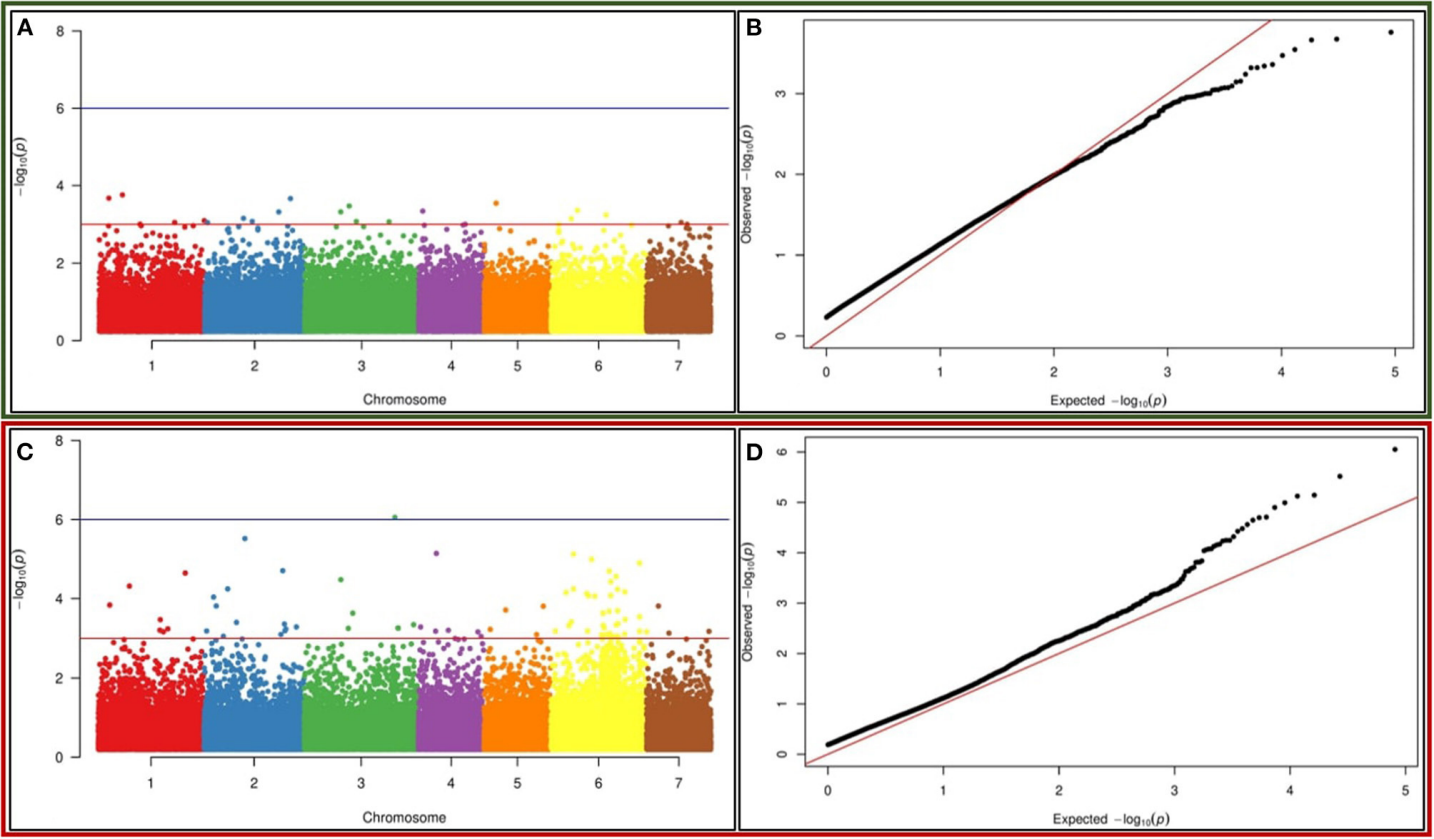
Genome-wide association studies (GWAS)-based Manhattan plots built in the TASSEL v5.2.64 environment exhibiting significant *p*-values measured by mixed linear model (MLM) model for **(A)** DPPH **(C)** FRAP activity using 67 K SNPs in pearl millet. The x-axis illustrates the relative density of *Pennisetum glaucum* reference genome-based SNPs physically mapped on seven chromosomes. The y-axis displays the –log10 *p*-value for the significant association of SNP loci for DPPH the trait. Quantile–quantile plot for **(B)** DPPH and **(D)** FRAP activity using the MLM model, built in the TASSEL v5.2.64 environment. The x-axis displayed the expected –log10 *p*-value and y-axis represented the observed –log10 *p*-value.

Marker trait association (MTA) analysis was performed for DPPH and FRAP activity-related trait using TASSEL. The MLM model-based association mapping showed 65 SNP markers associated with DPPH, whereas 153 SNPs showed association with FRAP trait at a *p*-value ≤ 0.001 (Supplementary Tables 4, 5). The MTA test resulted from the MLM model were further visualized into Manhattan plots in which each SNP was plotted against their chromosomal positions and the observed *p*-values (on a –log 10 scale) to remark the highly significant SNP markers associated with a trait. Thus, higher stringent threshold values were considered to filter out the highly associated markers with a specific trait –log 10 (*p*-value = 3.0) to minimize the effects of moderate size of the association panel and background noise of the Manhattan plots. Twenty-one SNP markers were found to be highly associated with the DPPH trait at *p*-value = 0.001, and Manhattan plot visualization revealed the high –log 10 *p*-value ranging from 3.0 to 3.76 (Figure 5A). The percentage of phenotypic variation ranged from 4.8 to 10.4% for the DPPH trait. These markers were found to be distributed on chromosomes 1, 2, 3, and 6 for DPPH, which had the highest *r*^2^ value. Out of 153, 80 SNP markers exhibited strongest associations with FRAP activity based on the MLM model at the *p*-value of 0.0001. Interestingly, 23 SNPs showed strongest associations with FRAP based on the MLM model at the range of *p*-values = 9.5731E−04 to 3.6325E−08 (Figure 5C). The strongest association of these SNPs for FRAP trait was also explained by the Manhattan plot and the –log 10 *p*-value ranges from 3.7–6.0. Maximum number of markers were distributed on chromosome 6 for this trait followed by chromosomes 1 and 2. Interestingly, the phenotypic variance ranged from 4.7 to 14.92% for these markers, and they were present on chromosomes 1, 2, and 6 for FRAP, which had the highest *r*^2^ value. QQ (quantile–quantile) plots displayed linear distribution when plotted against the observed and expected distribution of *p*-values for both the traits (DPPH and FRAP) (Figures 5B,D).

A GLM model-based association mapping approach was also applied to identify the associated markers for comparison purposes. It found that 122 SNPs exhibited close association with DPPH and 388 SNP markers with FRAP at *p*-value ≤ 0.001, but only common markers present in MLM-based analysis have been considered for further downstream analysis (Supplementary Tables 4, 5). After filtering, four SNP markers were found to be highly associated with DPPH at –log 10 *p*-value ranging from 4.0 to 4.4 (Supplementary Figure 6). The phenotypic variance associated with them ranged between 4.4 and 10.4%, and they were distributed on chromosomes 1 and 6. Similarly, 60 SNP markers displayed strong association with FRAP at a very high confidence statistical power. The Manhattan plot showed that the –log10 *p*-value scores in all cases were very high (3.0–7.4) (Supplementary Figure 8). QQ (quantile–quantile) plots displayed a linear distribution when plotted against the observed and expected distribution of *p*-values for both the traits (DPPH and FRAP) (Supplementary Figures 7, 9).

### Identification of Candidate Genes

Furthermore, we investigated the candidate gene falling in the association region, which showed strong association with the trait of interest. The significant SNPs identified as a result of the GWAS were located by position on their respective chromosome of *Pennisetum glaucum* by reference genome assembly (https://cegresources.icrisat.org/data_public/PearlMillet_Genome/). A total of 436 gene surrounding each SNP was identified using the GFF file of the reference genome. A BLAST search against the NCBI database using Blast2go revealed a large number of hits, representing a vast number of candidate genes of which the functions included defense against biotic/abiotic stress factors, growth, development, and regulation of bioactive metabolites (Supplementary Figure 10). Out of 436, 18 genes, showed similarity with antioxidant biosynthetic pathway-related genes (Table 3). Functional annotation revealed that these genes contained molecular active sites involved in regulating antioxidant activities. For example, GDSL esterase/lipase (Pgl_GLEAN_10037404) was found to be involved in hydrolase activity and especially acting on ester bonds to promote the sequestration and accumulation of carotenoids. This suggests that these genes may be acting as co-factors in these pathways, or they may regulate genes through various chemical pathways to promote the accumulation of antioxidant compounds. Furthermore, some candidate genes were found to be directly associated with antioxidant biosynthetic pathways across all SNP datasets including UDP-glycosyltransferase 73D1-like (Pgl_GLEAN_10001315), agmatine coumaroyltransferase-2 (Pgl_GLEAN_10010945), anthocyanin 5-(6^‴^-hydroxycinnamoyltransferase) (Pgl_GLEAN_10025805), GDSL esterase/lipase (Pgl_GLEAN_10037404), purple acid phosphatase 15 (Pgl_GLEAN_10028864), DUF3755 family protein (Pgl_GLEAN_10026569), and ATP binding cassette (Pgl_GLEAN_10005798), which were deemed to be the most significant (P = 6.84E−06). These candidate genes will be taken forward for further verification by haplotyping and characterization of their functions in future studies.

**Table 3:** List of candidate genes residing around SNP markers found to be associated with DPPH and FRAP using generalized linear model (GLM) and mixed linear model (MLM) analysis on a collection of 222 individuals of the PMiGAP.

**Associated SNP**	**Candidate gene**	**Chr**	**Start**	**End**	**Strand**	**Functional annotation**
S3_254044945	Pgl_GLEAN_10001315	3	254,071,667	254,073,151	+	Flavanone 7-O-beta—glycosyltransferase
S6_180998971	Pgl_GLEAN_10005798	6	180,991,308	180,999,267	+	ATP binding cassette
S5_130817729	Pgl_GLEAN_10014356	5	130,814,880	130,815,485	-	Pathogenesis-related protein 1-like
S3_253451484	Pgl_GLEAN_10018369	3	253,448,279	253,448,695	-	NAC transcription factor
S2_34036202	Pgl_GLEAN_10025805	2	34,004,192	34,006,162	+	Anthocyanin 5-(6^‴^-hydroxycinnamoyltransferase)
S6_63963146	Pgl_GLEAN_10037383	6	63,946,271	63,948,373	+	Flavone 7-O-beta—glycosyltransferase
S3_230848548	Pgl_GLEAN_10010555	3	230,844,116	230,845,301	+	NAC domain containing protein
S1_42630463	Pgl_GLEAN_10009255	1	42,655,782	42,657,214	+	Flavanone 7-O-beta—glycoside
S7_131386682	Pgl_GLEAN_10024630	7	131,376,401	131,382,239	-	Kinase family protein
S5_153744160	Pgl_GLEAN_10009510	5	153,742,622	153,748,266	-	ATP binding cassette
S4_135618402	Pgl_GLEAN_10019136	4	135,631,750	135,633,026	+	Flanoid O-methyltransferase-like protein
S2_22074367	Pgl_GLEAN_10010945	2	21,992,330	21,994,014	-	Agmatine coumaroyltransferase-2
S6_84647706	Pgl_GLEAN_10019153	6	84,447,652	84,449,835	-	Putative DUF594 domain containing protein
S6_162685647	Pgl_GLEAN_10026569	6	163,032,154	163,035,976	+	DUF3755 family protein
S2_4439224	Pgl_GLEAN_10027118	2	4,490,408	4,491,589	+	Kinase family protein
S6_151551492	Pgl_GLEAN_10028864	6	151,435,588	151,435,863	-	Purple acid phosphatase 15
S6_146769180	Pgl_GLEAN_10037404	6	146,718,522	146,720,598	+	GDSL esterase/lipase
S4_136907468	Pgl_GLEAN_10012466	4	136,793,025	136,800,340	-	Glutathione S-transferase

## Discussion

Phytochemical antioxidants have numerous nutritional benefits, especially phenolic compounds, which are an important group of secondary metabolites with bioactive properties (e.g., hydroxycinnamates and flavonoids) and play a significant role in plants and human health conditions such as cancer, diabetes, and heart disease (Chandrasekara and Shahidi, 2011; Ofosu et al., 2020). Pearl millet [*Penisetum glaucum* (L) R. Br.] is widely cultivated as a dietary staple food in the arid and semi-arid regions of the world, particularly in India and Africa and known to be a good source of natural antioxidants. In the present study, we explored the antioxidant activity in the pearl millet germplasm (PMiGAP) set of 222 genotypes, by screening it for DPPH and FRAP assays. These assays are widely applied in numerous studies to investigate the free radical scavenging ability and for measuring antioxidant activity of natural extracts [Alam et al. (2013), López et al. (2014), and Berwal et al. (2016)]. The phenotypic evaluation and comparison of antioxidant activity in PMiGAP revealed distinct differences among pearl millet lines for antioxidant activities. The majority of pearl millet accessions were found to have higher FRAP activity. On an average, DPPH scavenging activity of 53.8% was observed among the pearl millet germplasm studied in this report. A similar trend of antioxidant capacity was also reported in other studies in pearl millet that demonstrated that antioxidant potential of pearl millet was greatly influenced by the cultivar (Fukumoto and Mazza, 2000; Pushparaj and Urooj, 2014; Kalteh et al., 2018). In five bran extracts of pearl millet, substantial levels of differences in total phenolics, flavonoids, and DPPH radical scavenging activities were reported (Iqbal et al., 2007). On a similar trend, Berwal et al. (2016) also reported a study on the antioxidant potential of 92 pearl millet genotypes by studying DPPH and ABTS radical assays.

Phenolic compounds such as phenolic acids and polyphenolic flavonoids are known to scavenge various free radicals by two different mechanisms, either by electron transfer or by hydrogen atom transfer, and protect cells and tissues of the body from oxidative stress, and thereby protecting humans from various diseases (Scalbert et al., 2005; Seifried et al., 2007; Valko et al., 2007). Flavones such as luteolin, apigenin, naringenin, and their o-methyl derivatives are reported to have anti-cancerous properties (de Morais et al., 2017; Salazar-Lopez et al., 2018). Interestingly, in our study, DPPH showed a positive correlation with AHP, luteolin (LUT), suggesting higher phenolic compound accumulation and increased antioxidant activities. A similar trend of correlation with LHDC and LDH was also observed for FRAP activities. The similar observations were also recorded by Awika et al. (2004), Dykes et al. (2005), and Shen et al. (2018) where ADH showed positive correlation with AP (*r*^2^ = 93^**^). This is consistent with the theory that the dihydroxy functionality present in the luteolin molecule increases, reducing power in comparison with flavonoids with a single hydroxy group on the B ring as found with apigenin (Leopoldini et al., 2004).

Association mapping has been successfully performed in a number of crops including wheat, rice, pearl millet, maize, and cotton (Srivastava et al., 2020). In pearl millet, genetic diversity of PMiGAP panel and GWAS has been combined recently for several agronomic traits and reported recently (Varshney et al., 2017). However, association analysis of antioxidant trait has not been reported in pearl millet so far and is being reported for the first time *via* this study. A total of 222 genotypes studied in this report were drawn from the PMiGAP, which represented global diversity for antioxidant-related traits in pearl millet. The origin of each of these genotypes is provided in Supplementary Table 1, confirming that they are highly variable and were collected from different parts of the world. Population structure analysis for these accessions revealed that these individuals were grouped into six clusters in the panel. Interestingly, our results corroborate with earlier studies; for example, Sehgal et al. (2015) reported that there were six subpopulations within these PMiGAP by using 345 entries of the PMiGAP and 37 SSR markers. Similarly, population genomic analysis was carried out by Kanfany et al. (2020) and reported that the pearl millet inbred lines derived from diverse geographic and agroecological features possessed five subgroups mostly following pedigree differences with different levels of admixture. They have also stated that high genetic diversity prevails in pearl millet, which is very useful in defining heterotic groups for hybrid breeding, trait mapping, and holds promise for improving pearl millet for yield and nutritional quality. Six sub-groupings of pearl millet were also observed by Serba et al. (2019) using 398 accessions and 82,112 SNPs.

In the present study, we report the significantly associated MTAs for the antioxidant-related traits using ~67 K SNPs. Thus, a total of 24 SNPs strongly associated with the antioxidant-related traits (qualified at –log10 *p*-value ≥ 3.0) were further searched for LD status and for candidate gene searching. GWAS of these was performed by using widely used statistical models GLM and MLM (Yu et al., 2006; Price et al., 2010). In association analysis, the GLM procedure takes into account the phenotypic and genotype data combined with population structure data, while MLM considers both population structure and kinship data (familial relatedness), and therefore considered a stronger method, which avoids the chances of finding spurious associations (Zhao et al., 2007). However, in MLM, sometimes, over-compensation with both Q and K may also lead to false negatives (type II error; Zhao et al., 2007, 2011; Anuradha et al., 2017). Therefore, to overcome such difficulties, in this study, both the statistical models (MLM and GLM) were applied for antioxidant association, and only the common significantly associated markers were considered to be more reliable. Like us, He et al. (2019) had also relied on common MTAs using GLM or MLM for target traits that have a normal distribution.

A total of 436 genes were identified around each SNP found to be associated with DPPH and FRAP activity. Eighteen candidate genes including UDP-glycosyltransferase, agmatine coumaroyltransferase-2, anthocyanin 5-(6^‴^-hydroxycinnamoyltransferase), GDSL esterase/lipase, purple acid phosphatase 15, DUF3755 family protein, ATP binding cassette, and putative DUF594 domain-containing protein were found to be directly associated with antioxidant biosynthetic pathways across all selected SNP datasets. These candidates were found in close vicinity of the SNPs identified in this study but would require further validation in the future before they can be deployed in breeding programs. For validation, one can use candidate gene-based association mapping using large populations, or functional characterization through RNAi, VIGS, etc. Identification of desirable alleles of these MTAs will further confirm their roles in antioxidant synthesis and ensure their efficient utilization in crop improvement programs. Similar approaches as we report here were also adopted by Luo et al. (2020) for association analysis with variation in tocopherol and tocotrienol content, and these authors found that out of 47 associated SNPs, seven SNP markers were mapped in LD blocks (*r*^2^ > 0.6), while 185 of the other SNPs were not mapped in LD blocks. Thus, they identified 88 genes, which were predicted to be transcription factors, including NAC domain transcript factors, and Myb domain transcripts. However, they selected EgHGGT (homogentisate geranylgeranyl transferase) for further analysis, which is involved in biosynthesis of tocotrienols and had higher expression levels in the mesocarp compared with other tissues. Antioxidant-associated candidate gene identification has previously been attempted by many researchers in maize and other cereal crops. In maize, association analysis was performed by Li et al. (2012) to identify SNP markers associated with phenotypic variation of vitamin E and found that an indel 85 kb upstream of the ZmVTE4 gene was detected and found to play a role in the expression of the ZmVTE4 gene. Similarly, association analysis was applied by Diepenbrock et al. (2017) to identify two chlorophyll biosynthetic enzyme genes, which were responsible for major variation in vitamin E content in maize, and they observed that HGGT and prephenate dehydratase had positive roles in phenotypic variation in vitamin E content. GWAS analysis was also performed by Varshney et al. (2017) in pearl millet using 3,117,056 SNPs on 20 agro-morphological traits, and strong association of the markers for GNP (grain number per panicle) trait was observed at chromosomes 1 and 5. Association mapping and significant allelic association for grain iron and zinc were also demonstrated by Anuradha et al. (2017) in pearl millet. Similarly, association mapping analysis was also performed by Saïdou et al. (2014) and Sehgal et al. (2015) for many agronomically important traits in pearl millet.

In conclusion, the present study explored the genetic architecture of antioxidant content in pearl millet for the first time using LD-based GWAS exploiting historical recombination in a natural germplasm population. The population used showed a wide range of variability for traits studied. Furthermore, resequencing data and quality phenotyping of DPPH and FRAP led us to the identification of highly significant SNP markers associated with antioxidant content. Such associations provided better insights into the genetic architecture of this very important trait in pearl millet. Candidate gene analysis in the significantly associated SNP region has identified potential candidates, which on further validation, will provide a great resource to select these important traits in pearl millet breeding programs.

## Data Availability Statement

The original contributions presented in the study are included in the article/Supplementary Material, further inquiries can be directed to the corresponding author/s.

## Author Contributions

RSY conceived and supervised the completed study. JT, DY, PG, AW, and RY contributed to the phenotyping experiments. CY analyzed the results and wrote the manuscript. RKS and RBS developed the HapMap used for GWAS in this study. All authors contributed to the article and approved the submitted version.

## Conflict of Interest

The authors declare that the research was conducted in the absence of any commercial or financial relationships that could be construed as a potential conflict of interest.

## Supplementary Material

Supplementary Figure 1 Principal Component analysis (PCA) depicting variation among phenotypic traits for antioxidant activities and phenolic compounds.

Supplementary Figure 2 Single nucleotide polymorphism (SNP) density on seven chromosomes of pearl millet. The x-axis shows the interval distance in Mb. Window size to calculate SNP density 1 Mb.

Supplementary Figure 3 Annotation of single-nucleotide polymorphisms (SNPs) against reference genome of pearl millet showing distribution of SNPs in intergenic, upstream in different genic regions, synonymous, and non-synonymous SNPs detected within the CDS region.

Supplementary Figure 4 Determination of optimum K using Evanno's method identified during population structure analyses.

Supplementary Figure 5 LD plot built in the TASSEL v5.2.38 environment zoomed in on regions of high LD on chromosomes. The squared correlation coefficient (*r*^2^) values are denoted by a color scale from white (0.0) to red (1.0) in the upper triangle. The *p*-values ranging from non-significant (0.01; white) to highly significant (<0.0001; red) are shown in the lower triangle.

Supplementary Figure 6 GWAS-based Manhattan plots built in the TASSEL v5.2.64 environment exhibiting significant *p*-values measured by GLM model for DPPH activity using 67 K SNPs in pearl millet. The x-axis represented by SNPs on seven chromosomes and y-axis exhibited the -log10 *p*-value for the significant association of SNP loci for both the trait DPPH.

Supplementary Figure 7 Quantile-quantile plot of DPPH using the GLM model, built in the TASSEL v5.2.64 environment.

Supplementary Figure 8 GWAS-based Manhattan plots built in the TASSEL v5.2.64 environment exhibiting significant *p*-values measured by GLM model for FRAP activity. The x-axis displayed the distribution of SNPs throughout all pearl millet chromosomes and y-axis represented the -log10 *p*-value for the significant association of SNP loci for FRAP.

Supplementary Figure 9 Quantile-quantile plot representing distribution pattern for SNP marker associated with FRAP trait along with *p*-value analyzed through GLM model.

Supplementary Figure 10 Functional annotation of biological processes of genes residing around traits (DPPH and FRAP) associated SNPs.

Supplementary Table 1 Details of the 222 accessions of the PMiGAP genotypes having origin from around the world and used in this study.

Supplementary Table 2 Sample details of the pearl millet genotypes used for HPLC analysis.

Supplementary Table 3 Phenotyping values of 222 PMiGAP pearl millet genotypes represented by mean values for FRAP assay and DPPH activity.

Supplementary Table 4 Significantly associated SNP markers with DPPH at *P* < 0.001 analyzed through MLM and GLM based statistical model on a collection of 222 individuals of the PMiGAP.

Supplementary Table 5 List of markers associated with FRAP trait at *P* < 0.001 using MLM and GLM based methods on a collection of 222 individuals of the PMiGAP.
