# Supplementary figures and images for: Identifying Anti-Oxidant Biosynthesis Genes in Pearl Millet [Pennisetum glaucum (L.) R. Br.] Using Genome—Wide Association Analysis
Source: Front Plant Sci. 2021 May 28;12:599649. doi: 10.3389/fpls.2021.599649 (PMC8194398; doi:10.3389/fpls.2021.599649)

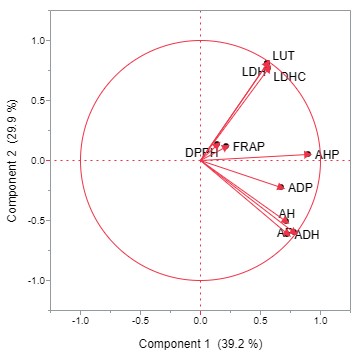

Supplement: Supplementary Figure 1 — Principal Component analysis (PCA) depicting variation among phenotypic traits for antioxidant activities and phenolic compounds. [file Image_1.JPEG]

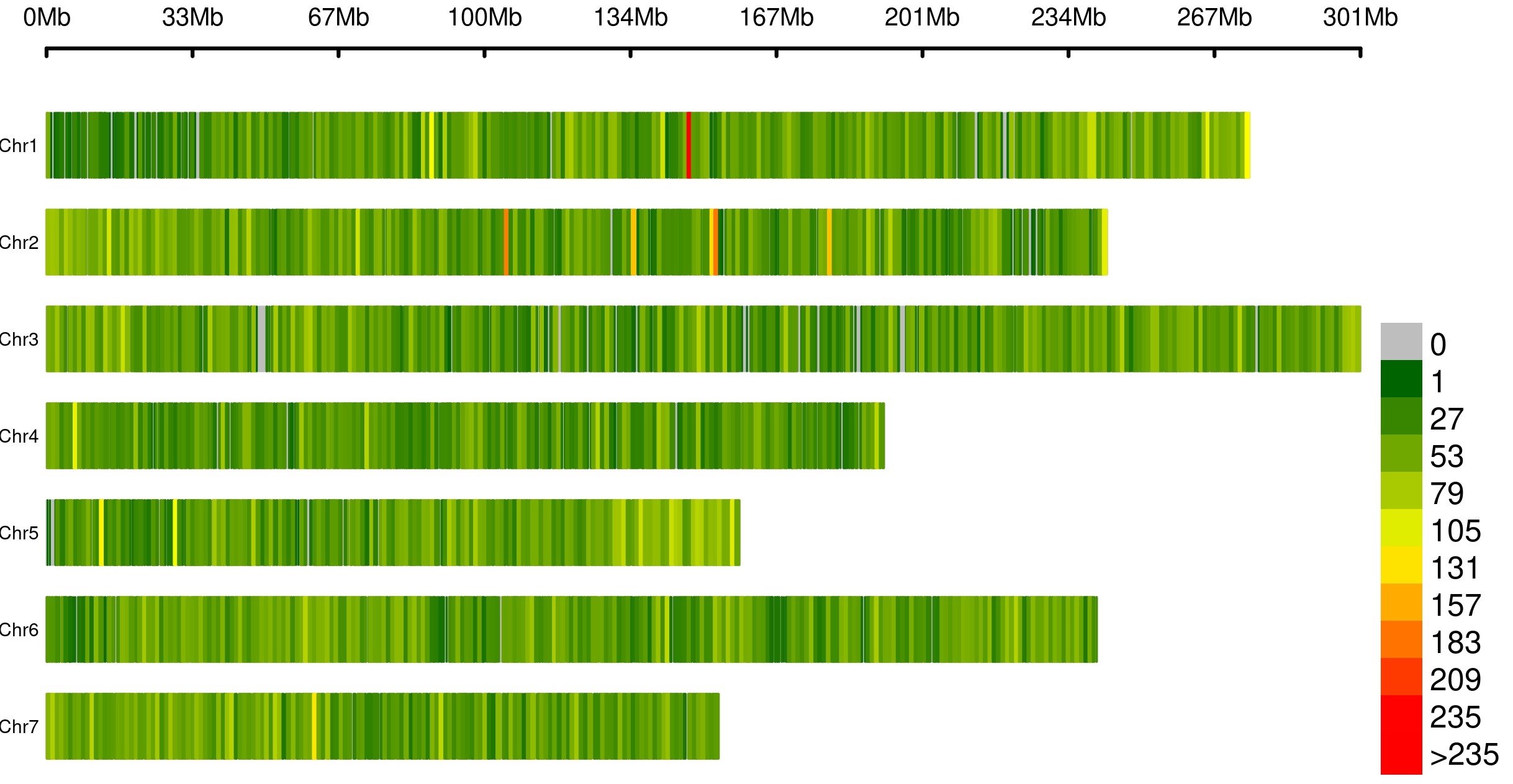

Supplement: Supplementary Figure 2 — Single nucleotide polymorphism (SNP) density on seven chromosomes of pearl millet. The x-axis shows the interval distance in Mb. Window size to calculate SNP density 1 Mb. [file Image_2.JPEG]

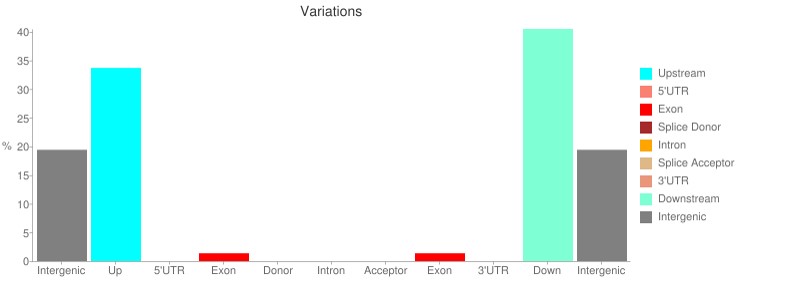

Supplement: Supplementary Figure 3 — Annotation of single-nucleotide polymorphisms (SNPs) against reference genome of pearl millet showing distribution of SNPs in intergenic, upstream in different genic regions, synonymous, and non-synonymous SNPs detected within the CDS region. [file Image_3.JPEG]

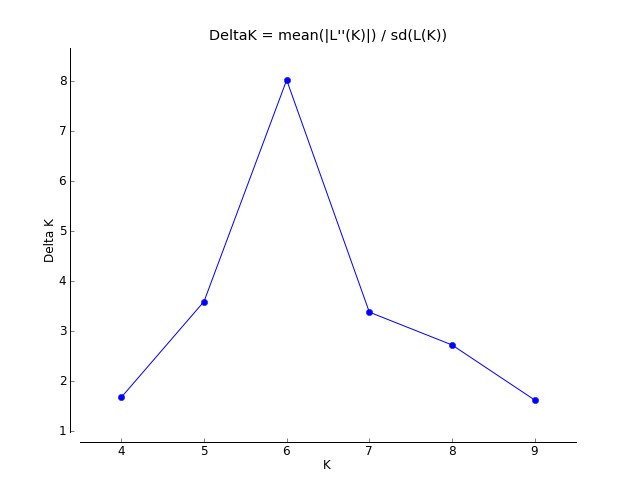

Supplement: Supplementary Figure 4 — Determination of optimum K using Evanno's method identified during population structure analyses. [file Image_4.JPEG]

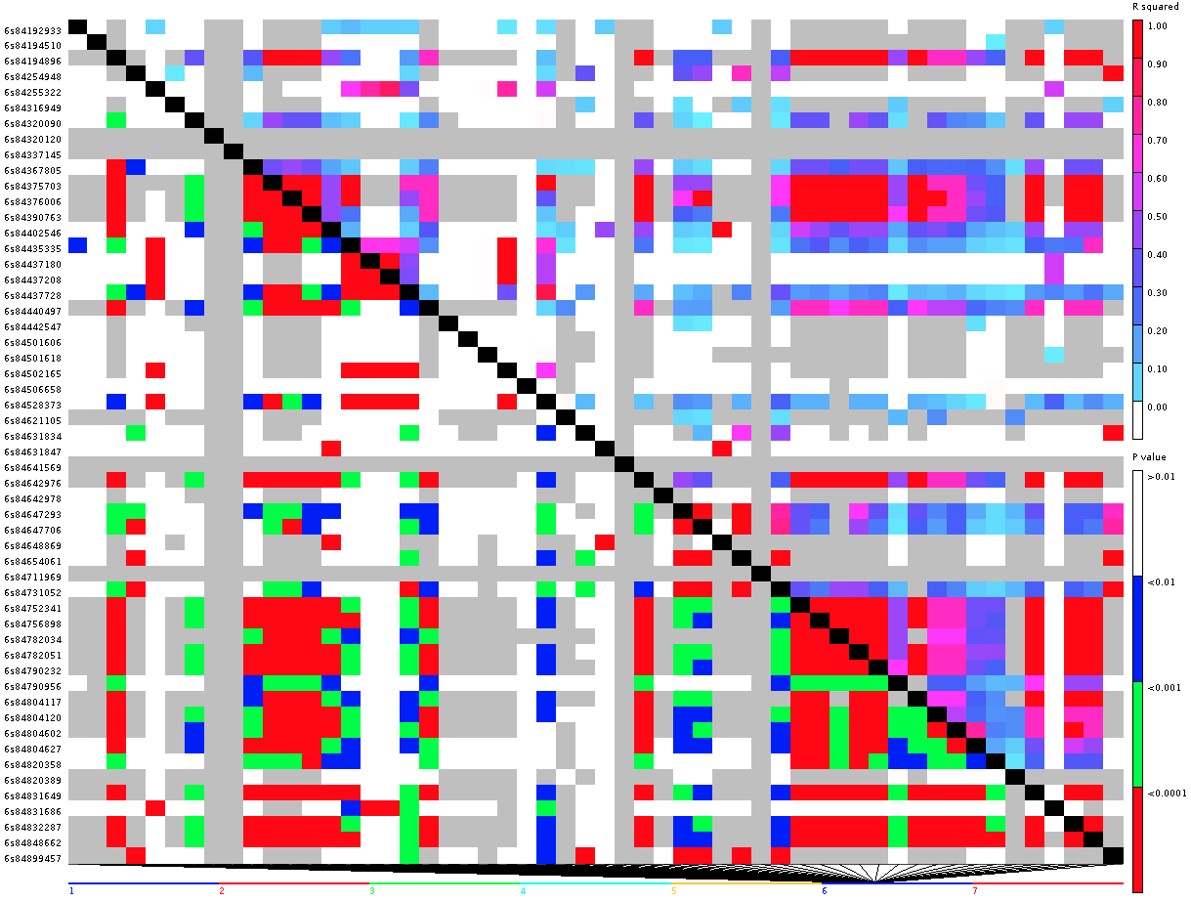

Supplement: Supplementary Figure 5 — LD plot built in the TASSEL v5.2.38 environment zoomed in on regions of high LD on chromosomes. The squared correlation coefficient (r2) values are denoted by a color scale from white (0.0) to red (1.0) in the upper triangle. The p-values ranging from non-significant (0.01; white) to highly significant (<0.0001; red) are shown in the lower triangle. [file Image_5.JPEG]

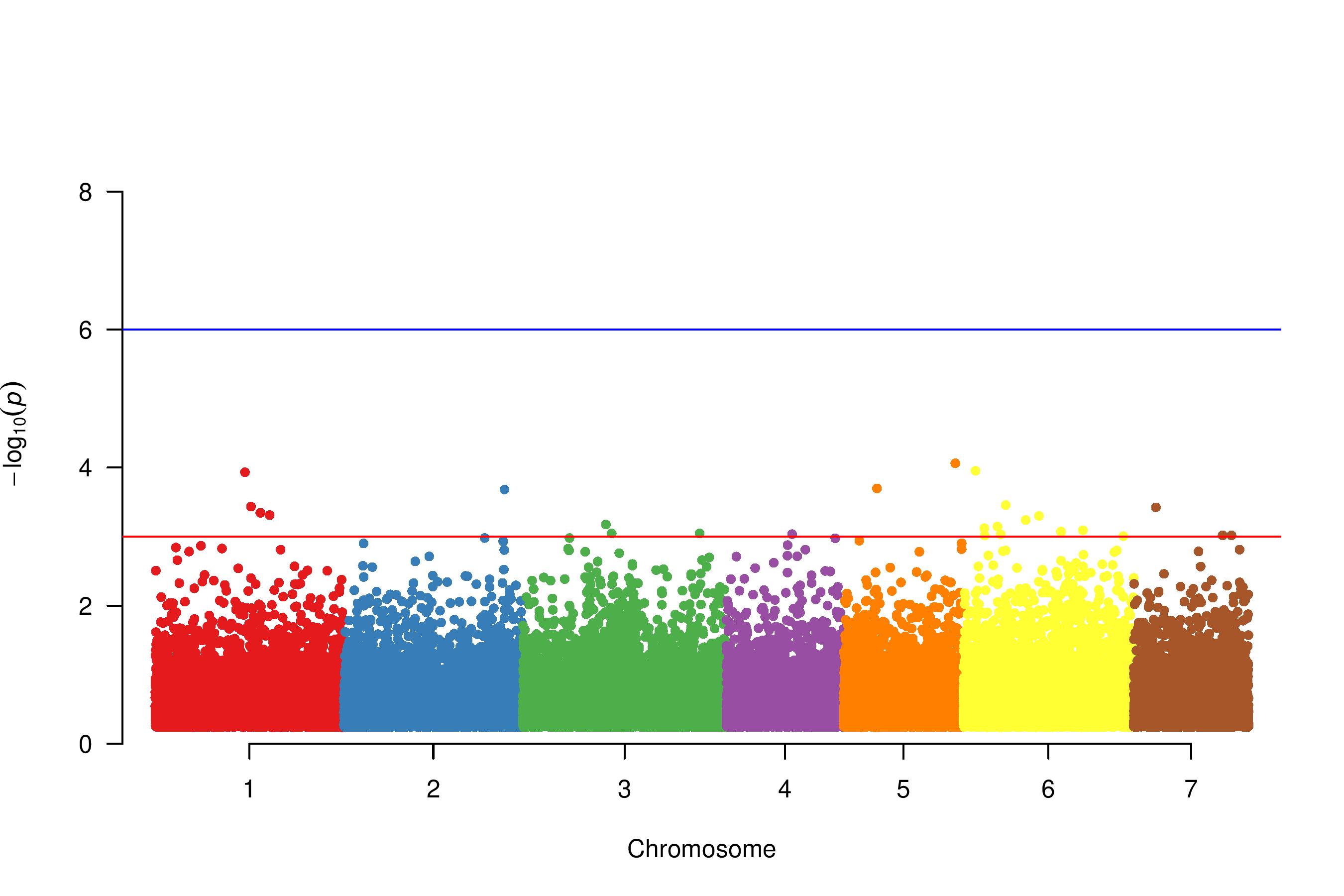

Supplement: Supplementary Figure 6 — GWAS-based Manhattan plots built in the TASSEL v5.2.64 environment exhibiting significant p-values measured by GLM model for DPPH activity using 67 K SNPs in pearl millet. The x-axis represented by SNPs on seven chromosomes and y-axis exhibited the -log10 p-value for the significant association of SNP loci for both the trait DPPH. [file Image_6.JPEG]

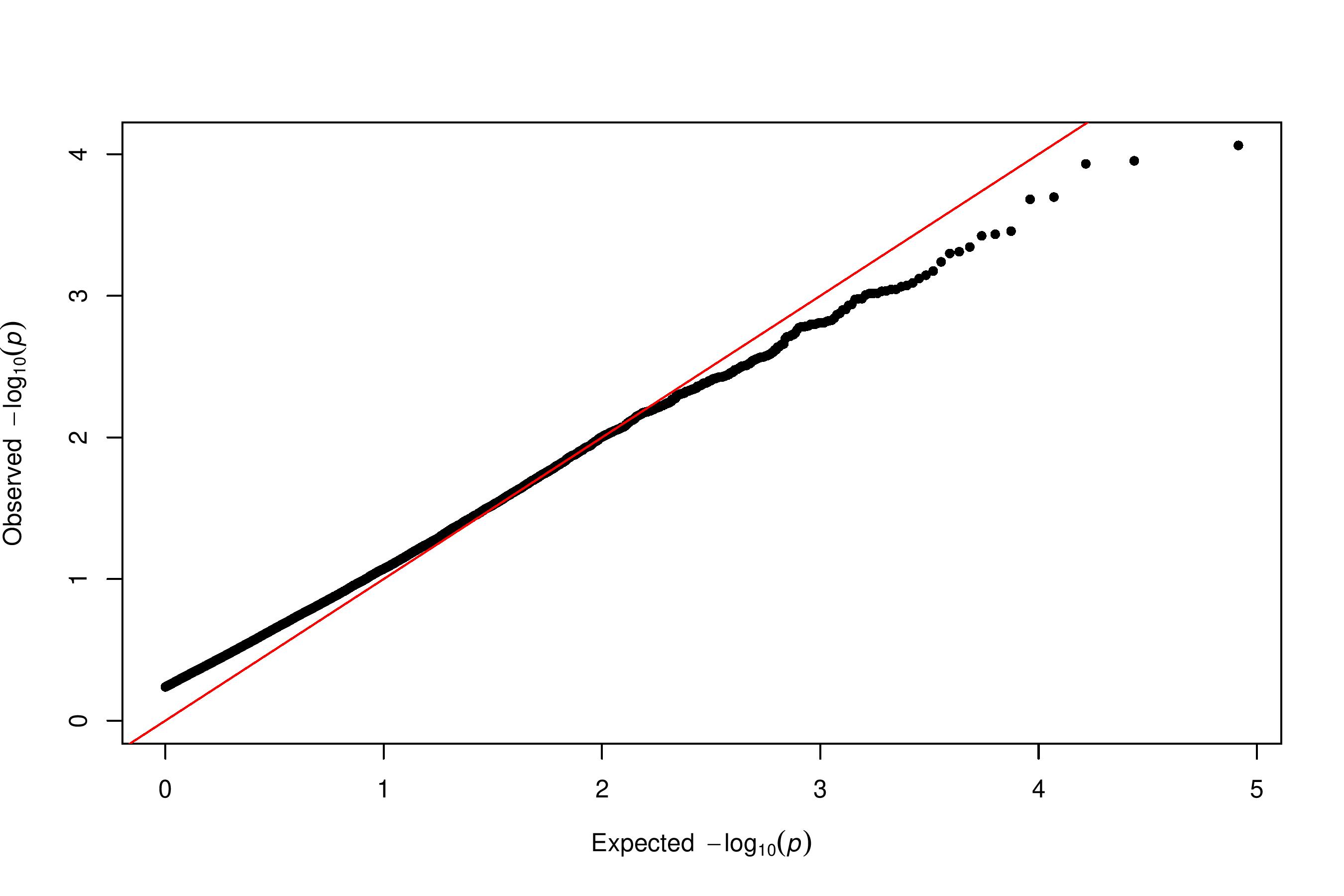

Supplement: Supplementary Figure 7 — Quantile-quantile plot of DPPH using the GLM model, built in the TASSEL v5.2.64 environment. [file Image_7.JPEG]

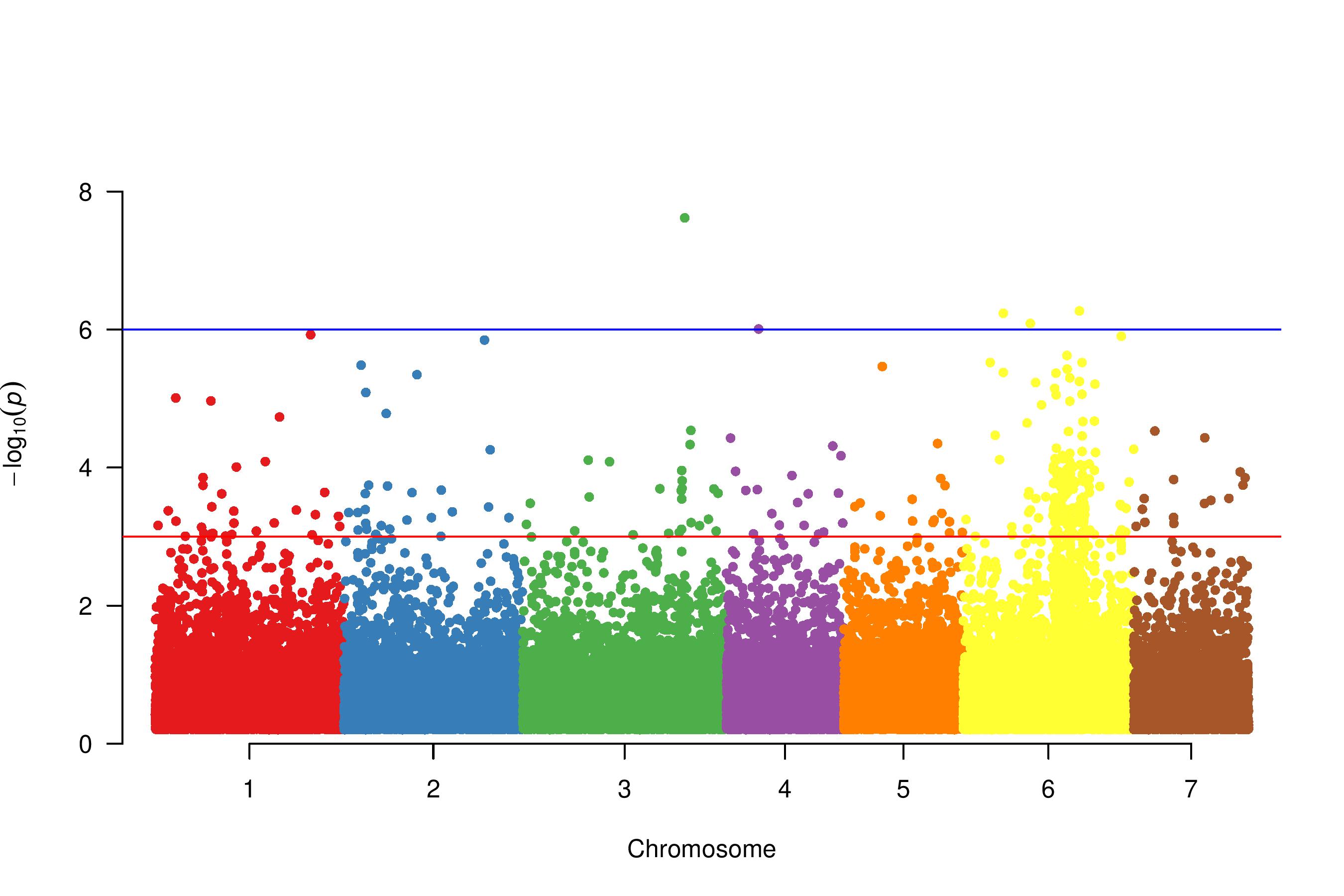

Supplement: Supplementary Figure 8 — GWAS-based Manhattan plots built in the TASSEL v5.2.64 environment exhibiting significant p-values measured by GLM model for FRAP activity. The x-axis displayed the distribution of SNPs throughout all pearl millet chromosomes and y-axis represented the -log10 p-value for the significant association of SNP loci for FRAP. [file Image_8.JPEG]

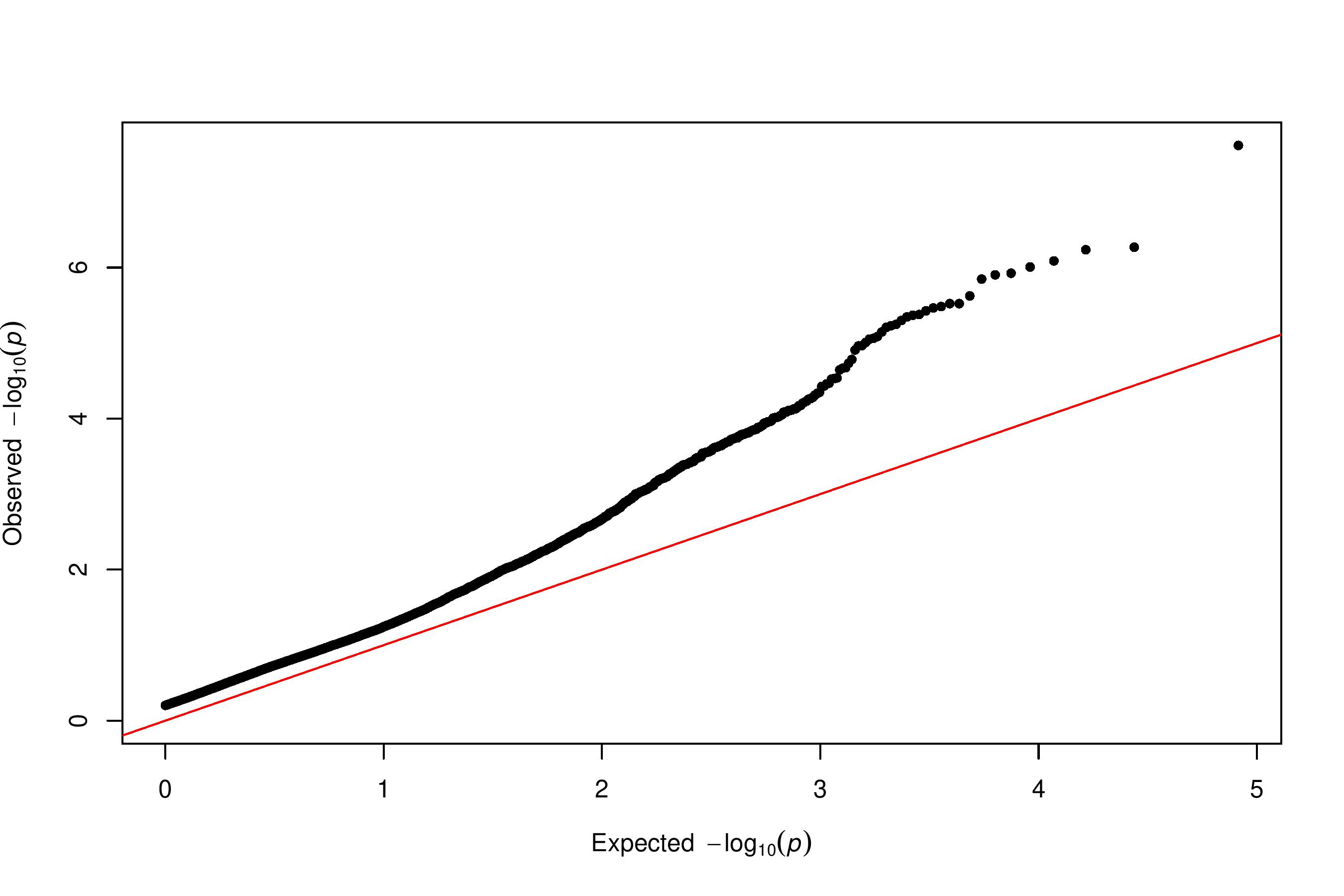

Supplement: Supplementary Figure 9 — Quantile-quantile plot representing distribution pattern for SNP marker associated with FRAP trait along with p-value analyzed through GLM model. [file Image_9.JPEG]

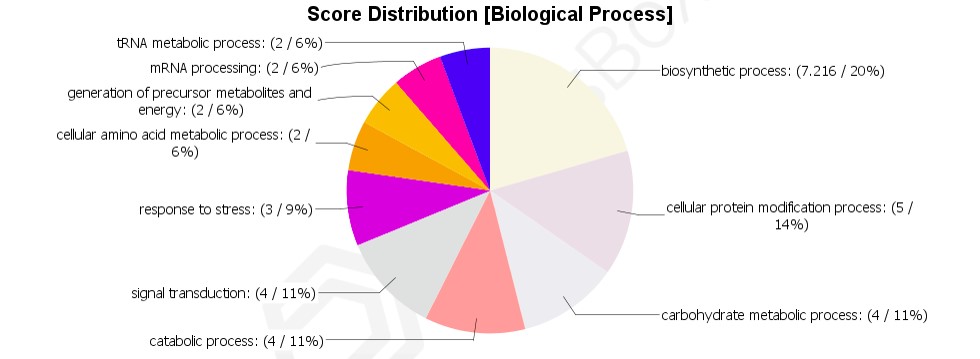

Supplement: Supplementary Figure 10 — Functional annotation of biological processes of genes residing around traits (DPPH and FRAP) associated SNPs. [file Image_10.JPEG]
